# Phosphatidylserine-Induced Conformational Modulation of Immune Cell Exhaustion-Associated Receptor TIM3

**DOI:** 10.1038/s41598-017-14064-x

**Published:** 2017-10-19

**Authors:** Jeffrey K. Weber, Ruhong Zhou

**Affiliations:** Computational Biology Center, IBM Thomas J. Watson Research Center, 1101 Kitchawan Rd, Yorktown Heights, New York, 10598 USA

## Abstract

In the face of chronic cancers and protracted viral infections, human immune cells are known to adopt an exhausted state in which their effector functions are lost. In recent years, a number of inhibitory receptors have been connected to the immune cell exhaustion phenotype; furthermore, ligands capable of activating these receptors have been discovered. The molecular mechanisms by which these ligands affect the exhausted states of immune cells, however, are largely unknown. Here, we present the results of molecular dynamics simulations of one potential exhaustion-associated system: the complex of human inhibitory receptor TIM3 (hTIM3) and its ligand phosphatidylserine (PSF). We find that PSF fundamentally alters the electrostatic environment within hTIM3’s Ca^2+^ binding site, facilitating the formation of a salt bridge and freeing a tyrosine-containing strand. This liberated tyrosine then collapses into a nearby hydrophobic pocket, anchoring a modified conformational ensemble typified by a β-strand rearrangement. The “electrostatic switching/hydrophobic anchoring” mechanism of conformational modulation reported here suggests a new type of process by which TIM3 activation might be achieved. This work also highlights strategies by which PSF-mediated conformational change could be controlled, either through administration of small molecules, execution of mutations, or modification of receptor phosphorylation states.

## Introduction

Immune cell exhaustion refers to a general state of cellular dysfunction in which, phenotypically, immunological effectors (such as T cells, B cells, and natural killer (NK) cells) lose their desired cytotoxicity in the face of chronic antigen exposure^1,2^. On transcriptional and posttranslational levels, exhaustion is typified by an aberrant expression and surface promotion of inhibitory receptors (iRs). These highly expressed iRs overregulate activating receptors (like T cell receptors) critical to immune response and ultimately bring about cell apoptosis. In cases of extended viral infection and cancer, immune cell exhaustion remains a central factor that adversely affects patient outcomes^1,2^.

In recent years, varied roles for the TIM (T cell immunoglobulin and mucin) family of receptors have been elucidated in immune cell regulatory pathways^3–15^. The three known human TIM proteins (hTIM1, hTIM3, and hTIM4) share the conserved features of an extracellular immunoglobulin V (IgV) domain, a mucin domain, a transmembrane domain, and a cytoplasmic tail^3–5^. With respect to the IgV domain, each of the hTIMs is characterized by two disulfide bonds that form the structural basis for a so-called FG-CC’ cleft bounded by its eponymous loops^3,6^. This cleft is known to be essential to ligation of TIM receptors, as discussed further below^3,5,9^.

The hTIM1 and hTIM4 receptors have been demonstrated to be costimulatory to the immune system, engaging in either intracellular interactions or heterophilic interactions with neighboring cells^3^. By contrast, hTIM3 is known to be inhibitory in nature^3–6^. The most studied process related hTIM3’s function concerns its engagement with the carbohydrate-binding protein galectin-9, which relies on the glycosylation of hTIM3 (potentially at both N- and O-linked sites) for recognition^6–8^. Interaction with galectin-9 plays a key role in moderating immune responses derived from helper T cells, inducing apoptosis where needed; attenuation of the galectin-9-induced hTIM3 response has been connected to an array of autoimmune disorders in humans^6–8^.

While galectin-9-related pathways indicate a potential therapeutic role for hTIM3 triggering^6^, TIM3 activation has also been associated with the phenotype of immune cell exhaustion^6,11–14^. Though galectin-9 may be involved in exhaustion^3,6^, accumulating evidence suggests that ligands other than galectin-9 interact with hTIM3 in a glycosylation-independent manner, and further that these other ligands play a part in establishing the exhausted state^11–14^. Despite recent findings that have cast doubt on whether (and, if so, how) the protein CEACAM1 binds directly to hTIM3, a clear functional role for CEACAM1 in T cell exhaustion has been established^13^. Other work on TIM3-based immune checkpoint blockade has also shown that TIM3 negatively regulates NK-cell function in a galectin-9-independent fashion, and suggests a potential role for the lipid phosphatidylserine (PSF) in immune cell exhaustion^15^.

PSF was first discovered to interact with the stimulatory members of the TIM family (hTIM1 and hTIM4), which, in complex with PSF, have a critical function in facilitating immune cell tolerance and preventing autoimmunity^9,10^. While typically concentrated on the inner envelope of lipid membranes, PSF becomes exposed on the outer envelopes of active and apoptotic immune cells. Interactions between hTIM1/hTIM4 and PSF are particularly important in the clearance of apoptotic cells, as stimulatory TIM/PSF complexes on macrophage surfaces are known to promote phagocytosis^9,10^. A phagocytic regulatory role for hTIM3/PSF complexes in macrophage and dendritic cells has also recently been identified^10^. It is unclear how hTIM3/PSF might impact effector cells. However, since PSF is involved in supporting immune cell tolerance – a downregulation of immune responses in the face of chronic antigen exposure – it is not unreasonable to hypothesize that PSF promotes the analogous process of immune cell exhaustion. Concerning the aforementioned work on reversing NK-cell exhaustion, de Silva *et al*. proposed that, since PSF is present on the surfaces of apoptotic cells, PSF might play a role in stimulating NK cell exhaustion after effector-induced tumor cell apoptosis^14^.

These intriguing questions about hTIM3/PSF interactions and their downstream effects beg an investigation of the protein structures and dynamics that give rise to hTIM3-associated signaling. Primary hTIM3 ligands are thought to bind to its extracellular IgV domain, initiating signal transduction pathways that pass through the membrane to control phosphorylation (across five tyrosine residues) of the hTIM3 cytoplasmic tail^3,6,15^. Many of the molecular details surrounding hTIM3’s activation and subsequent signaling pathways remain to be elucidated. However, understanding precisely how PSF binds to the hTIM3 IgV domain (and identifying the modified protein conformations that result) represents an important step for revealing potential mechanisms of hTIM3-mediated exhaustion.

In this work, we present the results of extensive molecular dynamics (MD) simulations of the hTIM3 IgV-PSF complex. Leveraging approximately 6 μs of aggregate simulation data initiated from an hTIM3 homology model, we found that PSF induces a dramatic conformational change in hTIM3 marked by (a) an expansion of the FG-CC’ cleft, (b) the formation of a strand-liberating salt bridge, and (c) the burial of a solvent-exposed tyrosine residue. The observed “electrostatic switching/hydrophobic anchoring” motif suggests a possible structural mechanism through which hTIM3 could be activated, highlighting a stable strand rearrangement in the IgV domain that could send signals downstream and perhaps facilitate changes in the phosphorylation state in the extracellular region of hTIM3.

## Models and Methods

### Homology Modeling

To date, no verified crystal structure of the hTIM3 IgV domain in complex with one of its activating ligands has been deposited. However, a structure for murine TIM3 (mTIM3) in complex with PSF has recently been solved^10^; in this case, PSF is bound directly to a Ca^2+^ ion found inside the FG-CC’ cleft of mTIM3. Given mTIM3’s high homology with hTIM3 (76% within the isolated extracellular domain) and PSF’s relatively small size, this structure lends itself well as a template for homology modeling and subsequent molecular dynamics simulation.

Therefore, a homology model for a 99-residue fragment of the apo-hTIM3 IgV domain (sequence: Fig. S1) was constructed based on this available murine template (PDB 3KAA^10^). An alignment between the two homologous sequence fragments is shown in Fig. S2. The best of ten structures (evaluated with DOPE score minimization) derived from the MODELLER v 9.1 software package^16^ was chosen for further study. The backbone configurations of all 10 structures derived from MODELLER are highly converged, however, suggesting later simulation results would be robust with respect to homology model choice (Fig. S3a). As expected, the chosen hTIM3 configuration is very similar to the mTIM3 template in structure (Fig. S3b). A Ca^2+^ ion was next placed into the FG loop of hTIM3 based on a simple alignment with the murine structure. Side chain and backbone configurations involved in the calcium binding site were optimized with a short minimization run *in vacuo*. Finally, a PSF molecule was inserted into the FG-CC’ cleft in alignment with the 3KAA structure, placing the phosphate group in direct contact with bound Ca^2+^ ion. This ternary complex was then subjected to another minimization.

### Molecular Dynamics Simulations

The resultant ternary homology model was next solvated in explicit TIP3P water along with 150 mM Na^+^ and Cl^−^ ions (intended to mimic physiological conditions), yielding a simulation system containing 18,192 atoms. The 60 × 55 × 60 Å simulation box was minimized for 10,000 steps and equilibrated with and without protein + ligand restraints in two respective 2 ns simulations. Production runs were conducted using the NAMD 2.10 simulation package^17–19^ under the NPT ensemble. Six parallel trajectories were extended to approximately 1 μs in duration, resulting in over 6 μs of aggregate simulation data. Temperature and pressure were controlled with a Langevin thermostat and a Parinello-Rahman barostat, respectively; parameters from the CHARMM36 force field^20^ were used for all simulated atoms, and SETTLE constraints^21^ were applied to water molecules.

As controls, MD data for two different apo hTIM3 complexes (one featuring just calcium bound, and the other featuring just PSF) and the PSF-bound mTIM3 complex (directly taken from PDB 3KAA) were also collected using procedures parallel to those listed above. The apo-PSF data were run until 6 μs of aggregate simulation time was reached; the apo-Ca^2+^ control was terminated after only 500 ns of data were collected, for reasons noted in the Results and Discussion. Only a single trajectory for the murine system was collected, but that trajectory was extended to 2 μs in length. Simulations demonstrate that the PSF-free, Ca^2+^-bound hTIM3 homology model is quite stable, undergoing no significant conformational change over the course of simulated dynamics (Fig. S3c,d). Results of the mTIM3 control simulations are discussed below in the context of holo-hTIM3.

### Markov Modeling

To synthesize the MD data drawn from parallel runs for both the ternary and apo-PSF hTIM3 ensembles, trajectories were combined into kinetic network models called Markov State Models (MSM)^22–28^. Using the MSMBuilder2 simulation package^29^, ternary complex configurations were clustered into 540 discrete states based on a protein backbone RMSD metric, where state radii were fixed at 2 Å. Transitions among these states were then counted according to a Markovian lag time window of 5 ns, a value deemed suitable after performing a heuristic relaxation timescale test (Fig. S4a). To further validate the final model, a Chapman-Kolmogorov test was performed on dynamics related to escape from the initial hTIM3 basin. A juxtaposition of dynamics drawn from the MSM and those derived from raw trajectories shows that the model, at this 5 ns lag time, does well in recapitulating the dynamics of the underlying MD data (Fig. S4b). Together, these tests indicate that our choices made in the course of MSM construction are reasonable. An analogous procedure was applied to construct a model for the apo-PSF hTIM3 dynamics; applying the same state radius and lag time yielded a model featuring 524 states. In each case, some extraneous states were trimmed to ensure ergodic network dynamics, and counts were symmetrized to enforce detailed balance. The final transition count matrices were normalized to yield *N* × *N* transition probability matrices^22^.

To simulate dynamics on the resulting MSM free energy surfaces, initial probability vectors localized in near-starting configurations were propagated according to the Chapman-Kolmogorov relation; these dynamics converge to an estimate of the equilibrium free energy surface in the long-time limit^22^. Dynamical free energies are simply defined as the negative logarithm of Markov state probabilities at a given point in time. Order parameter values were computed from 10 conformations drawn randomly from each Markov state, with probabilities distributed uniformly across these structures. In order to analyze heat dissipation in MSM trajectory ensembles, the λ-ensemble technique was invoked as described elsewhere^27,28^. In this case, dissipative dynamical free energy changes are defined as negative log-ratios of probabilities in dissipative (λ = 2) and unbiased (λ = 1) states.

With the exception of results related to the mTIM3 control, all dynamics discussed below are drawn from these MSMs constructed from the raw trajectory data. Notably, the aggregative power of MSMs allows for the description of time scales longer than any individual input trajectory^22^; here, our description of dynamics reaches a 5 μs time scale, though individual trajectories are only 1 μs in length.

## Results and Discussion

**Figure 1:**
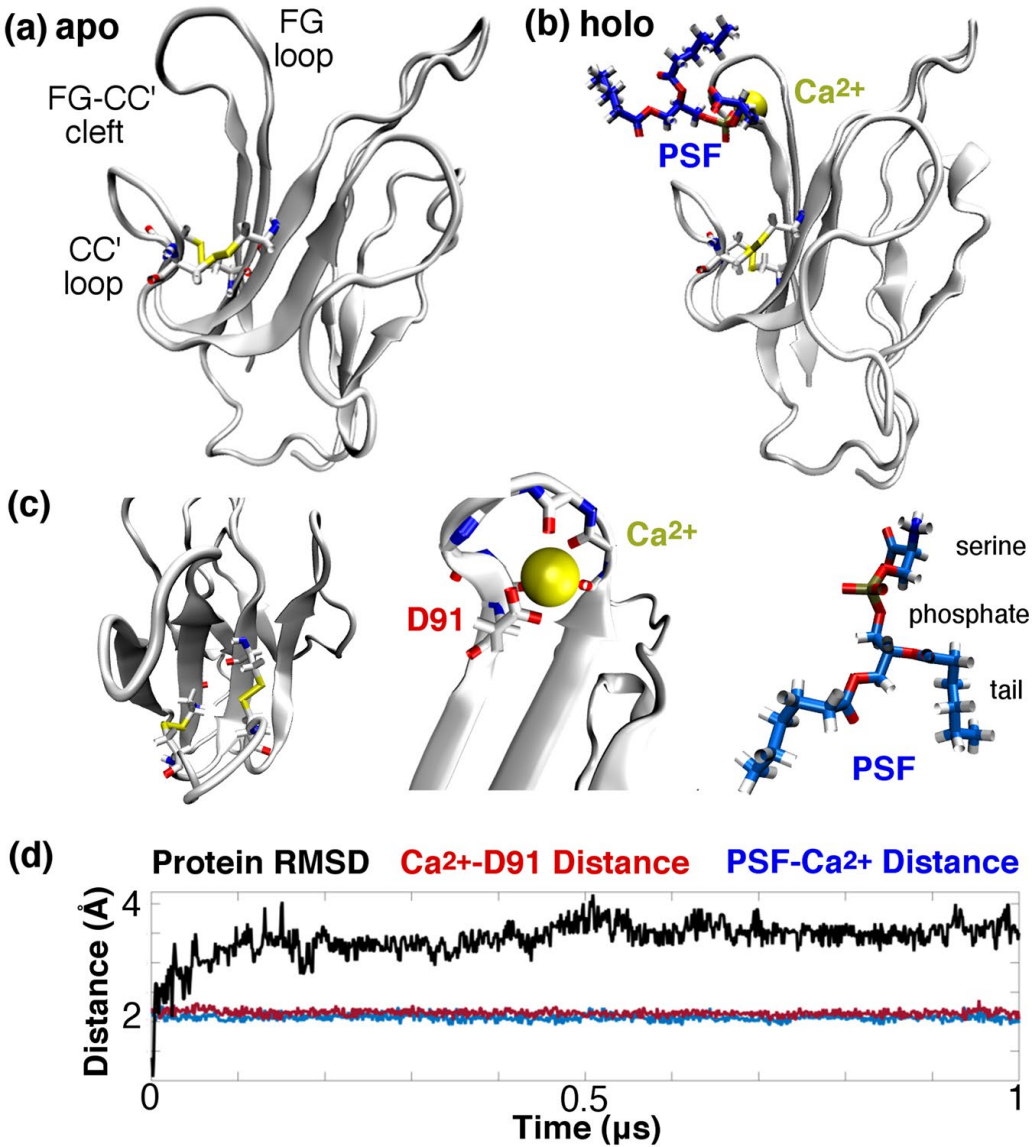
(**a**) Representation of hTIM3 apo state homology model featuring the putative FG-CC’ binding cleft. (**b**) Holo hTIM3- Ca^2+^-PSF complex with Ca^2+^ and PSF inserted into the FG-CC’ cleft. (**c**) Left: Top view of the FG-CC’ cleft, highlighting TIM3’s two noncanonical disulfide bonds in relation to the immunoglobulin superfamily. Middle: Illustration of Ca^2+^ binding site and chelating groups from TIM3. Right: Rendering of the phosphatidylserine (PSF) ligand, which contains zwitterionic phosphoserine and hydrophobic tail moieties. (**d**) Representative hTIM3- Ca^2+^-PSF dynamics illustrating the persistence of PSF binding and the extent of conformational change that results from PSF association. Protein RMSD is measured as a function of backbone atoms only.

As noted above, the hTIM3 homology model (Fig. 1a–c) features the FG-CC’ cleft characteristic of the TIM receptor family; the FG and CC’ loops are constrained by two disulfide linkages that are noncanonical within the immunoglobulin superfamily^3^. Based on the murine co-crystal structure^10^, one expects both Ca^2+^ and PSF to bind inside this FG-CC’ cleft (Fig. 1b). The putative Ca^2+^ binding site found in the homology model consists of five chelating oxygen atoms: three contributed by the protein backbone, and two donated by a negatively charged aspartate side chain.

The activating ligand PSF is a relatively common cell membrane lipid consisting of a zwitterionic serine group, a central phosphate moiety, and two alkyl chain tails (Fig. 1c)^10^. Both the phosphate group and serine carboxylate group are negatively charged and primed for interaction with the neighboring calcium ion. The alkyl tails of the PSF molecule are located in close proximity to the hydrophobic F32 of the CC′ loop.

Drawing a sample 1 μs trajectory from the hTIM3-Ca^2+^-PSF MSM, one can see that both PSF and Ca^2+^ remained quite stably bound within the FG-CC’ cleft over the course of simulation (Fig. 1d). The calcium ion stayed in tight contact with D91, while the PSF molecule remained closely associated with the bound calcium ion. Despite the stability of this complex, we observed that hTIM3 underwent a significant conformational change upon PSF binding: the protein backbone RMSD shifted by approximately 4 Å over the course of the 1 μs of dynamics, as the trace in Fig. 1d indicates.

**Figure 2:**
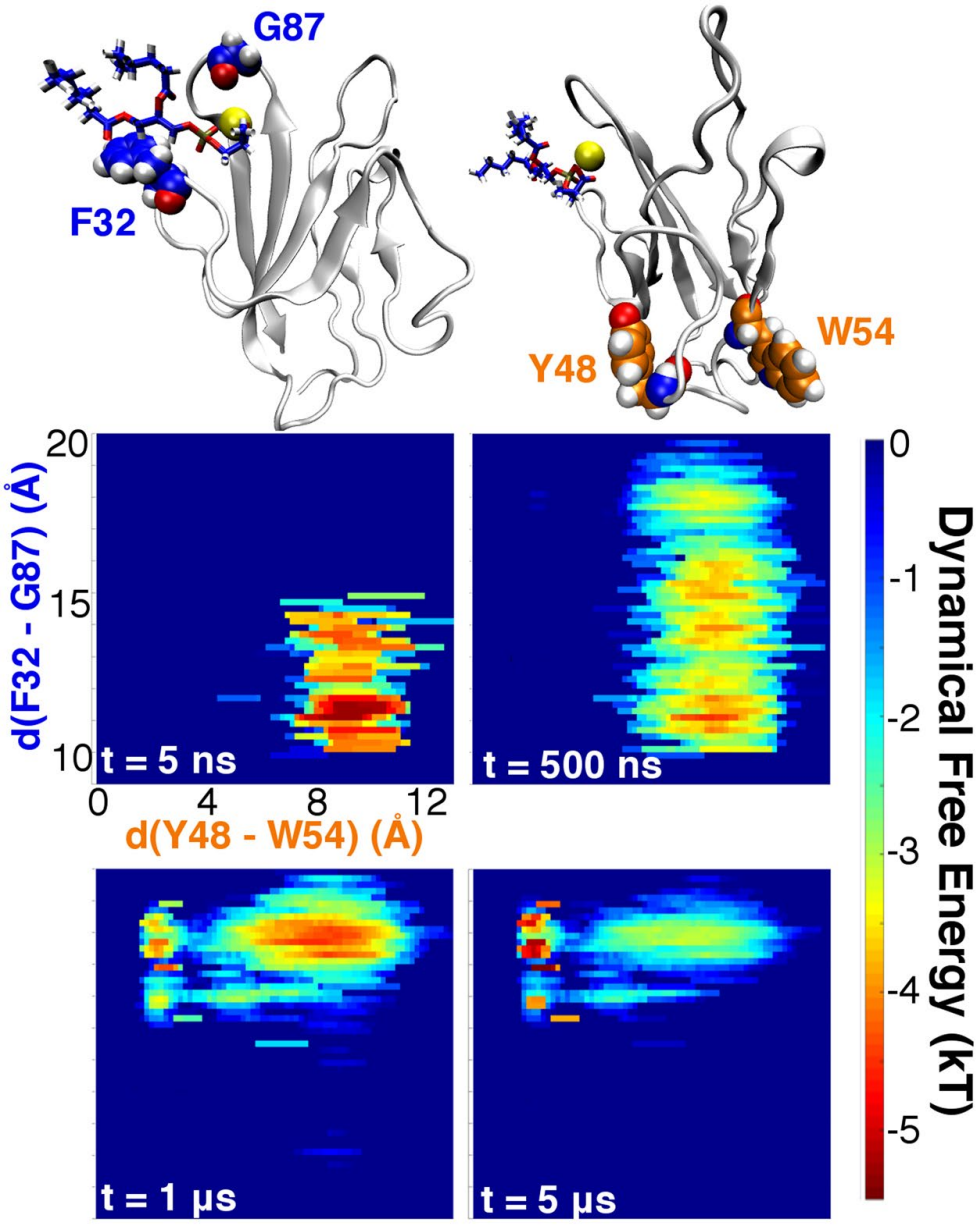
Conformational landscape of hTIM3-Ca^2+^-PSF as a function of time. Order parameters correspond to the opening of the FG-CC’ cleft (F32–G87 C_α_ separation) and sequestration of Y48 (Y48–W54 side chain separation). Dynamical free energies converge to an estimate of the equilibrium free energy in the long-time limit.

This PSF-induced conformational change can be well described as a function of two order parameters (Fig. 2): 1) the separation of the FG and CC’ loops, and 2) the distance between key hydrophobic residues Y48 and W54. Initializing dynamics in conformations close to the initial homology model (t = 5 ns), one notes that the FG-CC’ cleft quickly expanded upon PSF binding (t = 500 ns). This transformation seemed to be facilitated both by the steric constraints imposed by the lipid and a change in chelation of the Ca^2+^ ion. With respect to chelation, the Ca^2+^ ion engaged with both the serine carboxylate and phosphate groups of PSF, causing partial dissociation of the calcium from FG loop backbone oxygen atoms and freeing the FG loop to flip upward. The cleft-opening dynamics converged by the 1 μs time point, giving rise to a stable intermediate state in which Y48 and W54 remain largely separated. Over the next few microseconds of dynamics, the intermediate state evaporated as Y48-W54 contacts became more likely; after 5 μs of time evolution, the system settled into a new free energy minimum in which the FG-CC’ cleft remains open and Y48 is buried in a hydrophobic pocket centered around W54. Effectively, these dynamics suggest a conformational shift in which the Y48-containing strand swings from contact with a PSF-proximal strand to a PSF-distal one.

**Figure 3:**
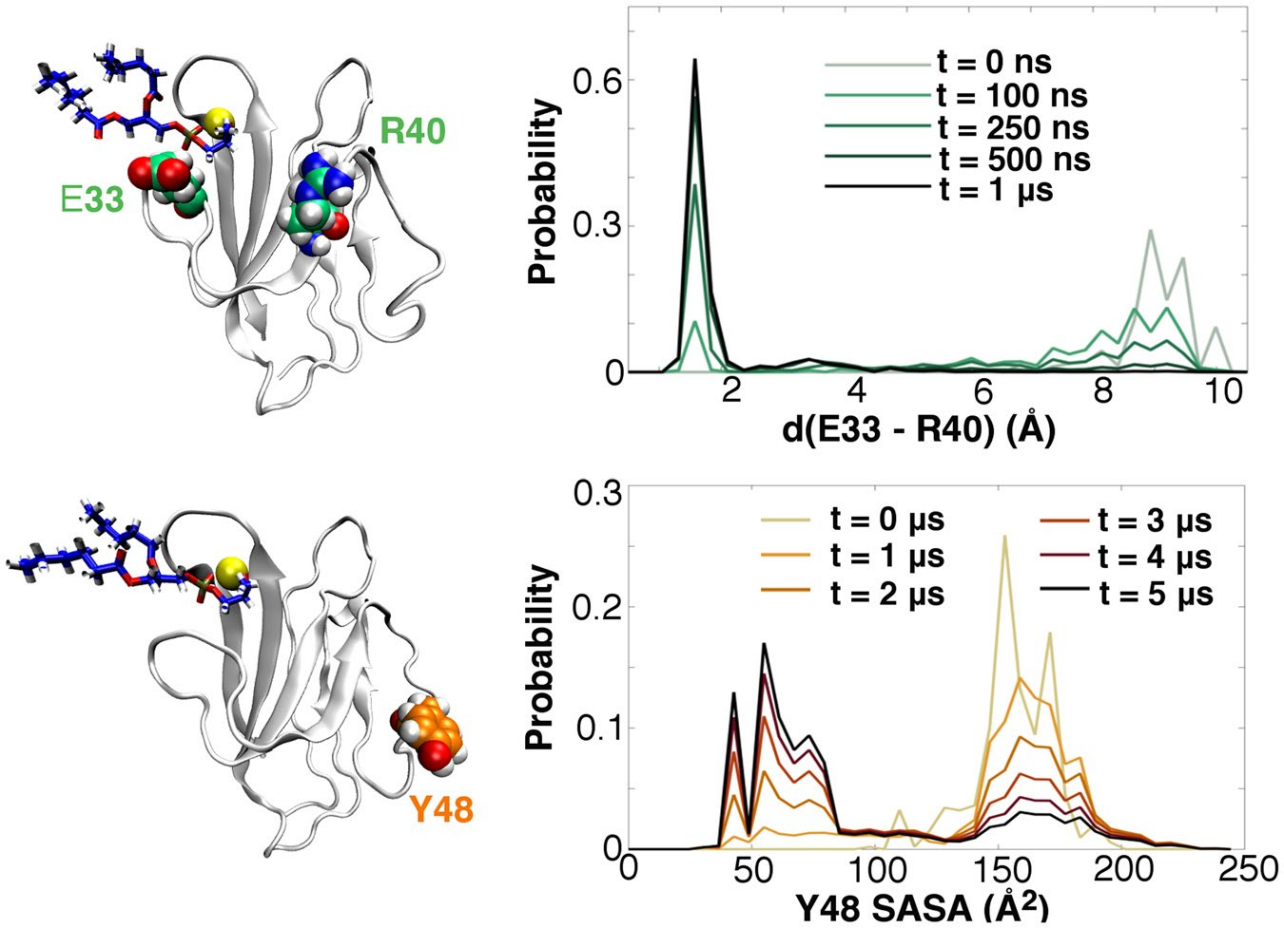
Intermediate salt bridge and Y48-strand dynamics contributing to PSF-induced conformational change in hTIM3. Top: Time evolution of the E33-R40 side chain distance. Bottom: Time evolution of Y48’s solvent accessible surface area. Notably, Y48 burial occurs on a much longer timescale than salt bridge formation.

The intermediate steps important to the initiation of this strand shift are shown in Fig. 3. Interestingly, though Y48-W54 binding was largely driven by the hydrophobic effect, the liberation of Y48 appeared to be controlled by an electrostatic switch. The Y48-strand is initially bound to the antiparallel strand containing R40; this arginine’s side chain starts at a relatively long distance (~1 nm) from a glutamate, E33, close to the FG-CC’ cleft. The presence of PSF imposed a significant change in the electrostatics of the binding site, as two negatively charged ligand moieties screened the Ca^2+^ ion from the rest of the protein. This electrostatic perturbation seemed to mediate attraction between the basic R40 and acidic E33 side chains, resulting in salt bridge formation. After 1 μs of dynamics, almost all probability density was localized in bound E33-R40 configurations. This salt bridge formation had the auxiliary effect of breaking an R40-N47 interaction, releasing the Y48-strand into solvent. Though solvent-accessible surface areas (SASAs) indicated Y48 is reasonably solvent exposed to start with, it is clear that Y48 becomes even more solvent exposed after its strand is freed (t = ~1 μs). As the dynamics progressed, however, an opposite effect dominated: Y48 was sequestered from solvent as it fell into the W54 hydrophobic pocket. Once the system had fully equilibrated (t = ~5 μs), Y48’s mean SASA was only approximately 25% of its initial value.

**Figure 4:**
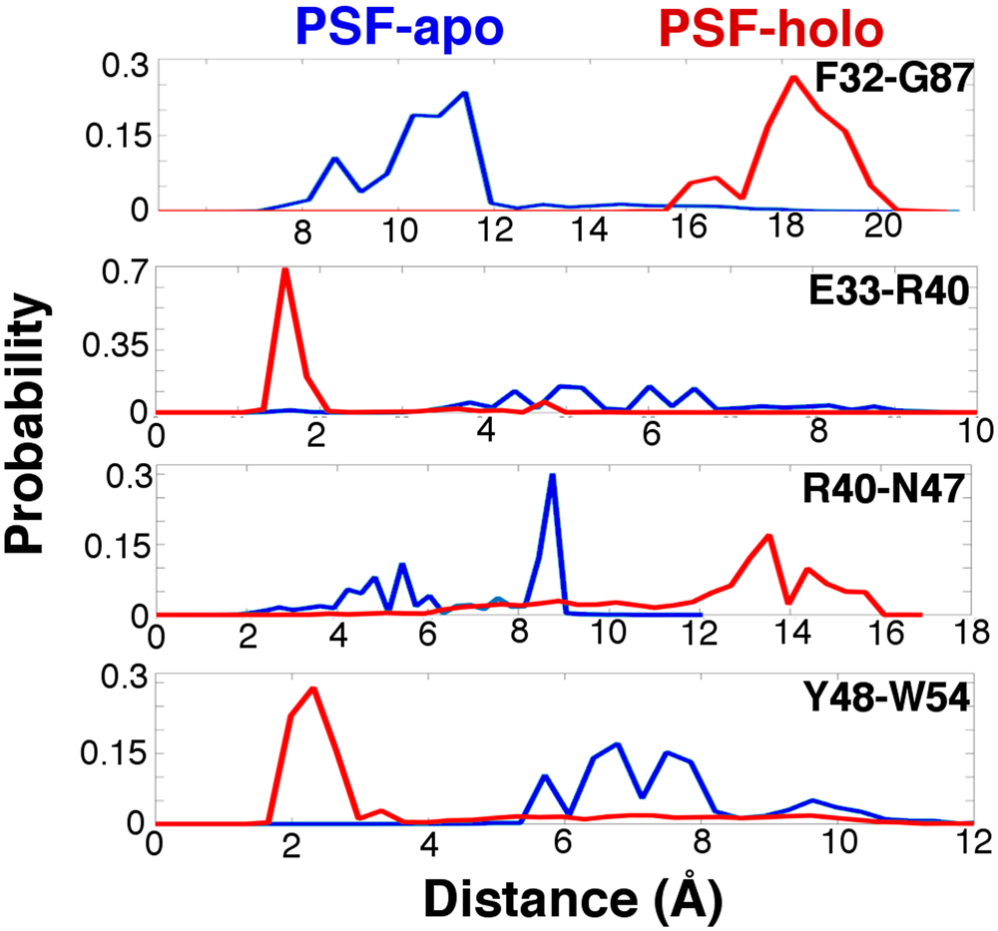
Comparison of equilibrium populations of Ca^2+^-bound hTIM3 in PSF-bound and PSF-free states. From top to bottom, the PSF-apo ensemble exhibits a preserved FG-CC’ cleft, a lack of salt bridge formation, a more stable, crystal-like strand pair, and a W54-distal Y48 with respect to PSF-holo conformations.

An important question then arises: does PSF really induce the observed conformational change? To verify this point, we simulated hTIM3-Ca^2+^ complexes in the absence of PSF binding as a control. The data shown in Figs 4 and S3 indicate that, indeed, the initial hTIM3 structure was almost completely conserved without PSF bound inside the FG-CC’ cleft. Specifically, without PSF, equilibrium probability distributions showed that (1) the FG-CC’ cleft remained closed, (2) E33-K40 did not form a salt bridge, (3) R40 and N47 remained (albeit somewhat transiently) bound, and (4) Y48 was not sequestered by W54 at all. While some fluctuations in the Y48-strand did occur over the course of 6 μs of PSF-apo simulations, the key conformational dynamics that typified the PSF-holo system did not occur at a level of significance without PSF binding. Since PSF binding has been reported to be Ca^2+^-dependent^6^, we also simulated hTIM3-PSF in the absence of calcium binding as another control. As expected, PSF quickly dissociated from the FG-CC’ cleft of hTIM3 under these conditions (Fig. S5).

**Figure 5:**
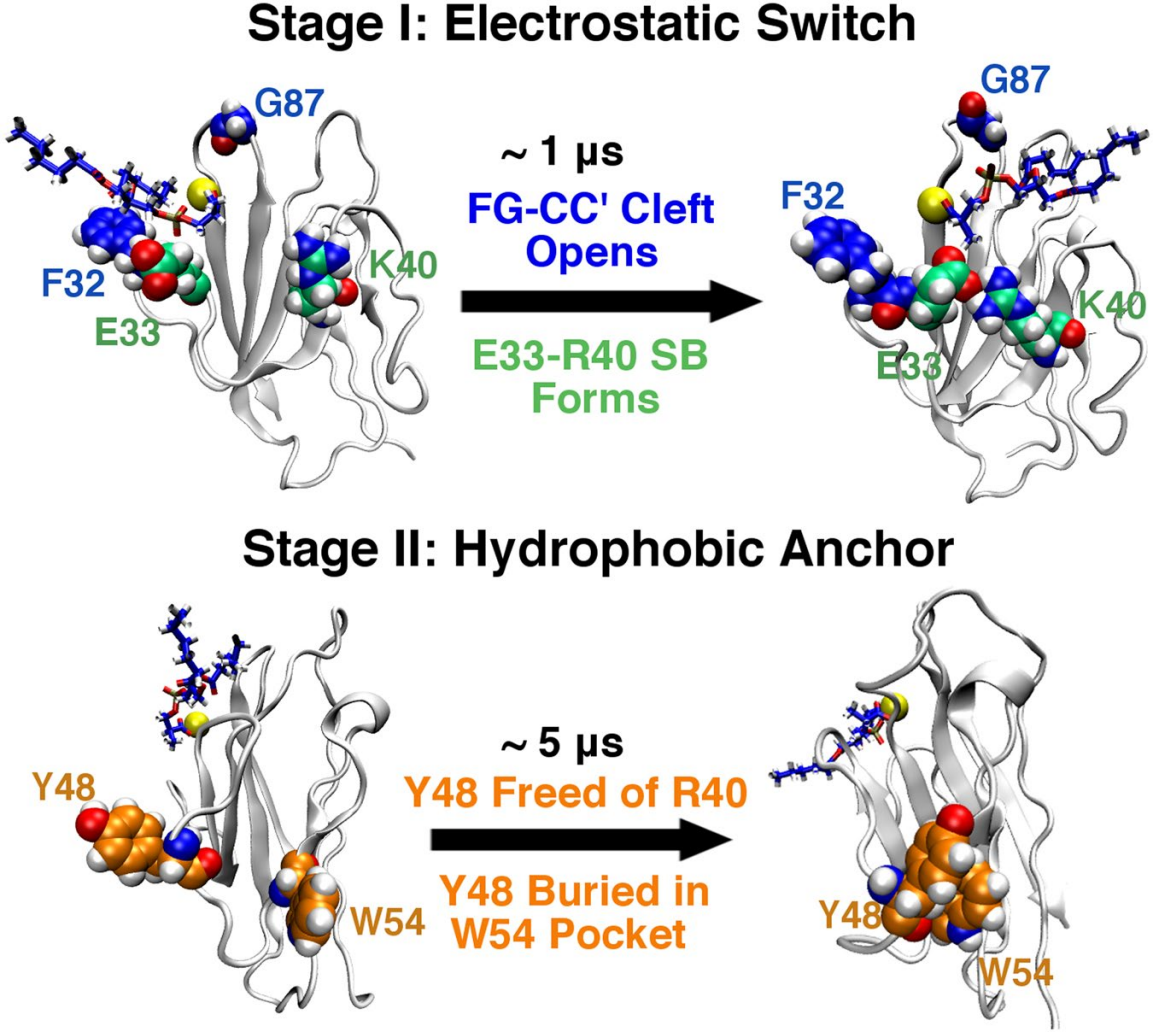
Proposed mechanism for PSF-induced conformational change in hTIM3, proceeding via two stages. Top: Stage I. Opening of the PSF binding cleft and formation of a E33-R40 salt bridge results in a free Y48 in approximately 1 μs. Bottom: Stage II. A solvent-exposed Y48 collapses into a hydrophobic pocket centered at W54 over an additional 4 μs of dynamics.

**Figure 6:**
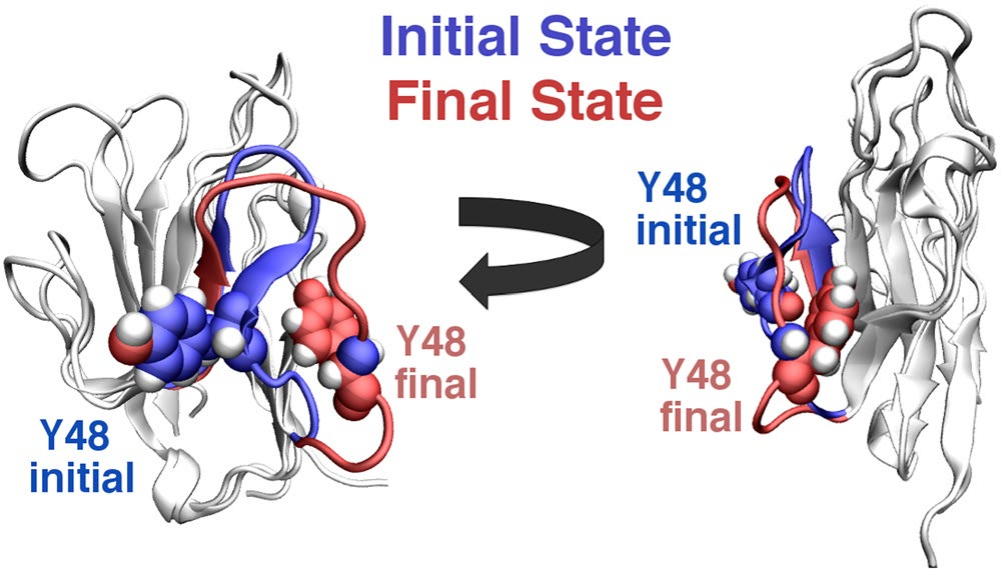
Overlay of representative initial and final hTIM3 conformations in response to PSF binding. The Y48-strand, highlighted in blue (initial) and red (final), shifts from contact with a PSF-proximal β-strand to a PSF-distal strand over the course of ~5 μs dynamics.

Based on the above observations, a sequential mechanism for the PSF-induced conformational modulation of hTIM3 has emerged. As Fig. 5 highlights, this process occurred in two stages. First, PSF binding had the direct effect of altering the electrostatics of the FG-CC’ cleft, allowing the FG/CC’ loops to separate, and facilitating E33-R40 salt bridge formation and Y48-strand liberation in concerted fashion. This approximately 1 μs-long stage culminated in the exposure of Y48 to solution, which then explored a range of configurations as part of a transient, flexible loop. A slower (5 μs) secondary process progressed after Y48 exposure: unable to reassociate with the now occupied R40, Y48 found a nearby hydrophobic pocket centered around W54. In particular, nanoscale dewetting (drying)^30–32^ provides a strong driving force for the burial of this hydrophobic tyrosine residue. The resulting Y48-buried, strand-shifted conformational state represented a new thermodynamic free energy minimum for PSF-bound hTIM3 that was very stable. This “electrostatic switching/hydrophobic anchoring” mechanism of conformational modulation is reminiscent of similar motifs seen in kinase activation^25^. Figure 6 provides structural superimpositions for the initial and final states of hTIM3 in response to PSF binding. As the overlaid structures demonstrate, the Y48-strand shifted from contact with a PSF-proximal β-strand (containing R40) to a PSF-distal β-strand (containing W54), in corroboration of the data in Fig. 2.

As a final control, we also collected 2 μs of simulation data related to PSF- and Ca^2+^-bound mTIM3, the template from which the hTIM3 homology model was built. The holo structure used to initiate the mTIM3 trajectory was pulled directly from the template PDB, 3KAA. Strikingly, we see that the Y48-strand shift noted in hTIM3 is not seen in mTIM3 dynamics (Fig. S6–S8). This observation is perhaps unsurprising from a crystallographic perspective, as our simulations confirm the stability of the deposited PSF-mTIM3 co-crystal structure. Several key points of sequence divergence between mTIM3 and hTIM3 hint at why this conformational change is only observed in the hTIM3 molecule. Specifically, moving from mTIM3 to hTIM3, the Q33E and Q55/W54 mutations give rise to the electrostatic switching/hydrophobic anchoring motif characterized in hTIM3. With glutamine at position 33, K40 is not driven as strongly to break its local contacts; E37 of mTIM3 (mutated to glutamine in hTIM3) also seems to hold K40 in place (Fig. S8). As such, no liberation of the Y48-strand is observed in mTIM3. Even if this strand were liberated in mTIM3, however, the presence of glutamine at position 55 (the homolog of W54 in hTIM3) suggests that the hydrophobic anchoring effect would be correspondingly weaker. Considered together, these factors provide a rationale for the stability of the PSF-mTIM3 crystal complex. Intriguingly, our results thus suggest a diverged PSF response (and perhaps a diverged signaling mechanism) between murine and human TIM3 molecules.

**Figure 7:**
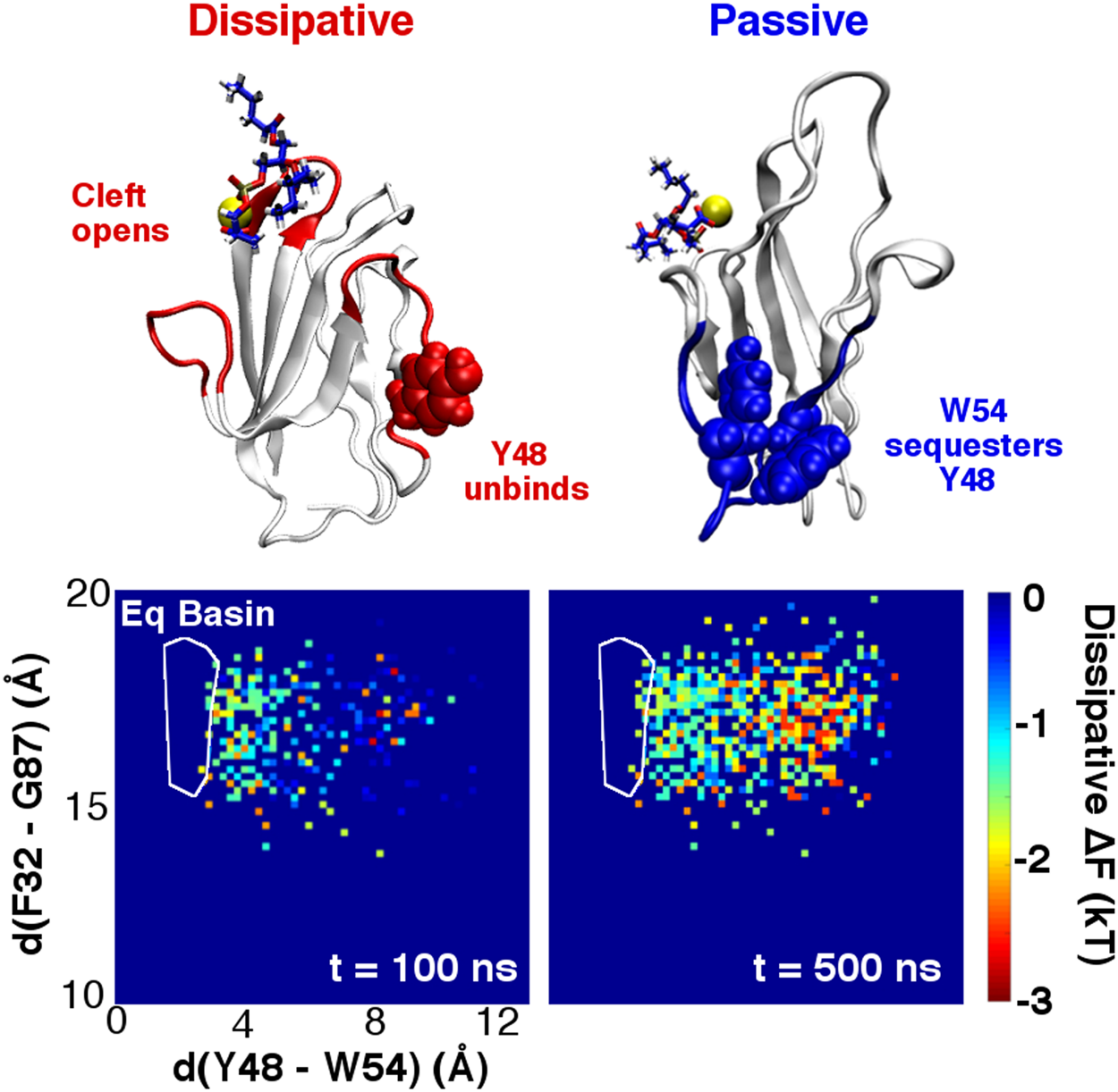
Passive and dissipative components of PSF-induced conformational change at 100 and 500 ns time points. Negative dissipative free energy changes correspond to probability increases within the dissipative trajectory ensemble. The white contours indicate the location of the dominant equilibrium basin seen in Fig. 2; dissipative dynamics occur almost entirely outside this basin, and largely involve the opening of the FG-CC’ cleft and unbinding of Y48.

Past simulation work has emphasized the connection between heat dissipation and signaling protein activation mechanisms^27^. Employing a similar analysis for hTIM3 (Fig. 7), we observed that the processes associated with cleft opening and Y48 liberation were quite dissipative in nature: hTIM3 states in which the FG loop is up and the Y48-strand is free experienced ten- to hundred-fold probability increases in dissipative trajectory ensembles probed at 100 ns and 500 ns time scales. However, the free energy minimum in which Y48-W54 was largely untouched by dissipative trajectories, suggesting that Y48 sequestration proceeded by a passive, near-equilibrium mechanism. These results suggest that direct PSF interactions (and the electrostatic perturbations they bring about) induced a thermodynamically irreversible transition into open-cleft and Y48-free states, a process that dissipated a significant amount of heat into the environment. Burial of Y48, however, then occured slowly and reversibly, producing little entropy in the process. This partitioning of passive and dissipative conformational events is also consistent with past observations of kinase and GPCR activation^25–27^, where fast, dissipative transitions were both seeded by and generative of passive conformational shifts.

Whether the conformational modulation detected in our simulations triggers hTIM3 activation remains an open question. However, the dynamics we did observe upon PSF binding (particularly the exposure and subsequent burial of a tyrosine) are notable in several regards. First, it is possible that PSF could control the activity of a tyrosine kinase at hTIM3-Y48. Conformationally-modulated tyrosine exposure is a well-known mechanism for modulating phosphorylation states in an array of signaling proteins^6,25,27^. While tyrosine kinases are most commonly found inside the cytosol, extracellular receptors are known to be extensively phosphorylated *in vivo*, and tyrosine kinases that are secreted and operate in the extracellular matrix have been recently discovered^33,34^. Given that the buried state of Y48 appears to be the thermodynamic native state of PSF-bound hTIM3, it is conceivable that PSF ligation could prevent some inhibitory/protective phosphorylation event at Y48, allowing TIM3 to be activated and stimulate exhaustion-related processes downstream. To our knowledge, no experimental evidence yet exists of Y48’s propensity for interaction with a tyrosine kinase; we feel this prospect does, however, warrant further experimental investigation.

It is more likely that this PSF-induced conformational change effects some additional change in the receptor molecule that exposes kinase-targeted tyrosines on hTIM3’s cytoplasmic tail. One classical result of surface-receptor ligation is dimer formation, an interaction scheme that is known to induce further conformational change at the dimer interface and impact cytosolic domain conformations^3^. Though TIM2 is indeed known to dimerize in mice, no evidence yet substantiates the hypothesis that TIM3 dimerizes upon PSF binding in any system^3,4^. It is possible, of course, that PSF simply induces some downstream conformational changes in isolated hTIM3 that make cytoplasmic tyrosine residues accessible to kinases. In either case, the present results suggest that hTIM3’s Y48 is essential for maintaining the initial conformational change brought about by PSF. In the design of strategies aimed at mitigating PSF-induced immune cell exhaustion — involving either small molecule- or mutation-based approaches — one might look to preventing the burial of Y48 as a preliminary goal.

Notably, only the extracellular IgV domain of hTIM3 is simulated in this work. The mucin, transmembrane, and cytoplasmic regions of hTIM3 are located on the coterminal side of the IgV domain; in most renditions of the IgV domain in this work, these additional domains would be found below the lower limits of the image. Just how the conformational change observed in the hTIM3 IgV domain impacts the structures of the mucin, transmembrane, and cytoplasmic domains remains an unanswered question. It is also noteworthy that the PSF known to associate with hTIM3 is thought to be derived from cell membranes, either from the cell’s own bilayer or from nearby apoptotic/broken cells^3,4,6^. The PSF-binding FG-CC’ cleft of the IgV domain is located some distance from the membrane surface^3,4,6^. Whether hTIM3 only binds PSF that is free in solution, or whether PSF can also migrate to hTIM3 and be subsequently culled from the membrane, also represent interesting topics for study.

## Conclusion

Combining homology models, MD simulations, and complex network descriptions of dynamical landscapes, we have elucidated the conformational changes brought about by PSF binding to the extracellular IgV domain of the receptor hTIM3. PSF association alters the electrostatic environment of the FG-CC’ ligand binding cleft conserved among TIM receptors, screening a bound calcium ion with two negatively charged groups and flipping the FG loop upward. This electrostatic perturbation draws a basic residue toward the binding cleft, facilitating the formation of a local salt bridge and freeing a tyrosine-containing strand in the process. After liberation, this tyrosine collapses into a hydrophobic pocket surrounding a distal tryptophan, anchoring a new strand-pair in place.

As noted above, it remains unclear whether this PSF-induced conformational change in hTIM3 represents a first step in a pathway toward immune cell exhaustion. It is nonetheless interesting that, based on structural homology and physical simulation techniques, one can show 1) hTIM3 forms a very stable complex with its activating ligand and 2) formation of this complex imparts a substantial and persistent conformational change on the extracellular protein domain. Based on these results, simulations of hTIM3 in complex with other activating ligands (such as HMGB1^14^, and perhaps CEACAM1^13^) are certainly warranted. In particular, one wonders if these other, larger ligands will evoke similar conformational changes via binding to the FG-CC’ cleft, or if other conformational mechanisms specific to protein-protein interactions will emerge. More distantly, one expects that atomistic simulations of the full hTIM3 protein (including its mucin, transmembrane, and cytosolic domains, traversing an explicit membrane segment) will become tractable, helping to illuminate the true functional implications of ligand binding^35^. Until that time, one hopes that the testable hypotheses generated by simulation work like that presented here – e.g., with regard to the role of Y48 in the PSF-activated complex of hTIM3–will help guide experimental explorations of the mechanisms of immune cell exhaustion.

## Electronic supplementary material

Supporting Information
