# Supplementary material for: Phosphatidylserine-Induced Conformational Modulation of Immune Cell Exhaustion-Associated Receptor TIM3
Source: Sci Rep. 2017 Oct 19;7:13579. doi: 10.1038/s41598-017-14064-x (PMC5648779; doi:10.1038/s41598-017-14064-x)
Supplement: Supplementary file 1 — Supporting Information [file 41598_2017_14064_MOESM1_ESM.pdf]

Supplementary Information: Phosphatidylserine-Induced Conformational Modulation of Immune Cell Exhaustion-Associated Receptor TIM3

Jeffrey K. Weber and Ruhong Zhou*

Computational Biology Center, IBM Thomas J. Watson Research Center, 1101 Kitchawan Rd, Yorktown Heights, New York 10598

*to whom correspondence should be addressed

As discussed in the main text, this supplementary document contains images and captions corresponding to Figs. S1, S2, S3, S4, S5, S6, S7, and S8.

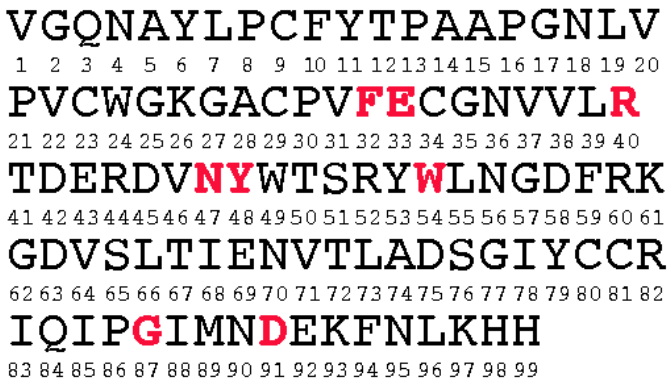

Fig. S1. hTIM3 IgV domain sequence fragment simulated in this work. Specific residues noted in the main text are highlighted in red.

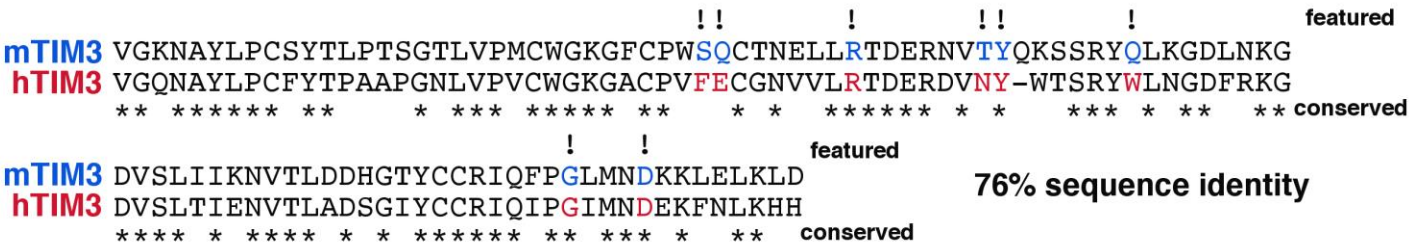

Fig. S2. Alignment between murine TIM3 (mTIM3) template and human TIM3 (hTIM3) target sequence fragments. Sequences are limited to the extracellular domain of TIM3, which are 100 residues (mTIM3) and 99 residues (hTIM3) in length. Conserved residues are marked by asterisks, and featured residues (shown in red in Fig. S1) are marked by exclamation points. In whole, the two sequences are 76% identical.

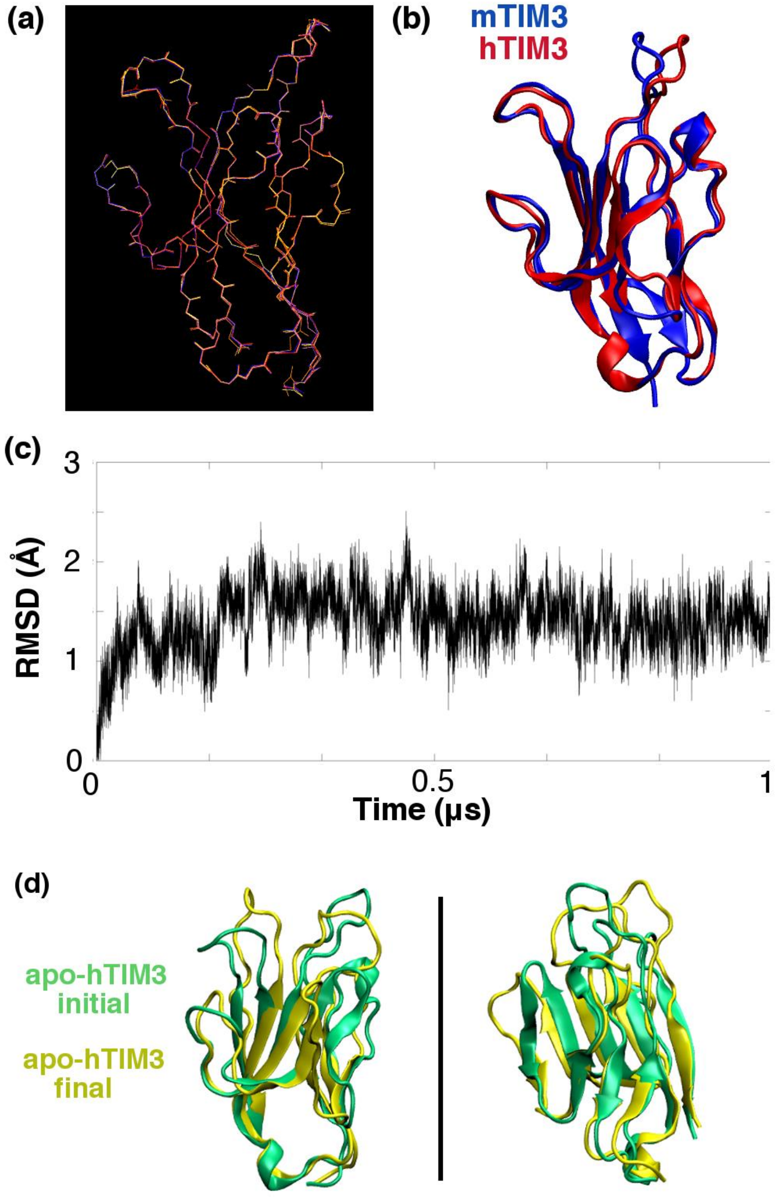

Fig. S3. Validation of the hTIM3 homology model used in the main text. (a) Overlay of 10 homology models derived from the MODELLER software package. Backbone configurations from these output models are highly converged. (b) Alignment of mTIM3 template and final hTIM3 homology model, illustrating the expected high degree of structural similarity between template and target. (c) RMSD of the Ca^{2+}-bound, PSF-free hTIM3 homology model over the course of 1 μs of MD simulation. In general, the RMSD remains around 1.5Å from its original structure. (d) Structural overlay of initial hTIM3 homology model and final configuration after 1 μs of simulation. Though loop positions fluctuate, no significant conformational change occurs over the course of simulation.

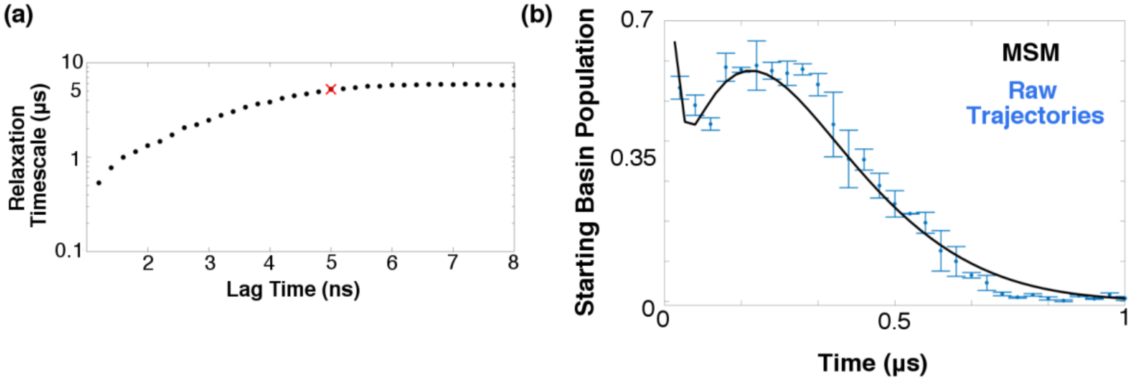

Fig. S4. Validation of MSM dynamics featured in main text. (a) Longest relaxation timescales as a function of lag time for the MSM of PSF-bound hTIM3 dynamics. Relaxation timescales begin to level at a lag time of approximately 5 ns, suggesting that value is appropriate for use in further model construction. (b) Results of Chapman-Kolmogorov test of MSM dynamics. Here, dynamics of escape from the initial hTIM3 basin (defined by a F32-G87 distance of less than 12.5Å, and a Y48-W54 distance of greater than 5Å) are compared between the raw 1 μs trajectories and those propagated by the MSM transition matrix. Error bars represent standard deviations for populations computed from the six 1 μs trajectories used in model construction. Overall, the MSM population at the chosen lag time decays at approximately the same rate as in the raw trajectories, and with the same line shape. These results suggest the MSM properly recapitulates the dynamics found in the underlying raw data.

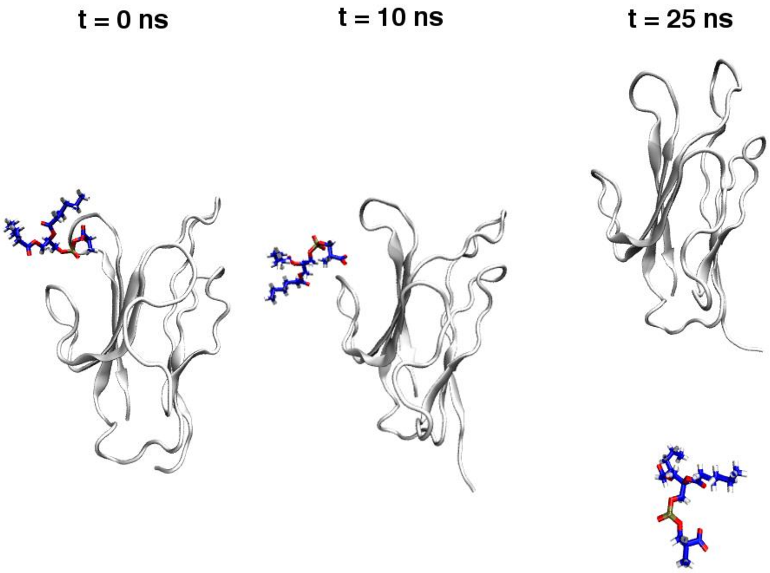

Fig. S5. PSF-hTIM3 complex simulations in the absence of calcium. As the three frames indicate, PSF quickly dissociates from the FG-CC' cleft of hTIM3 without the stabilizing effect of the positively charged ion bound to the FG loop.

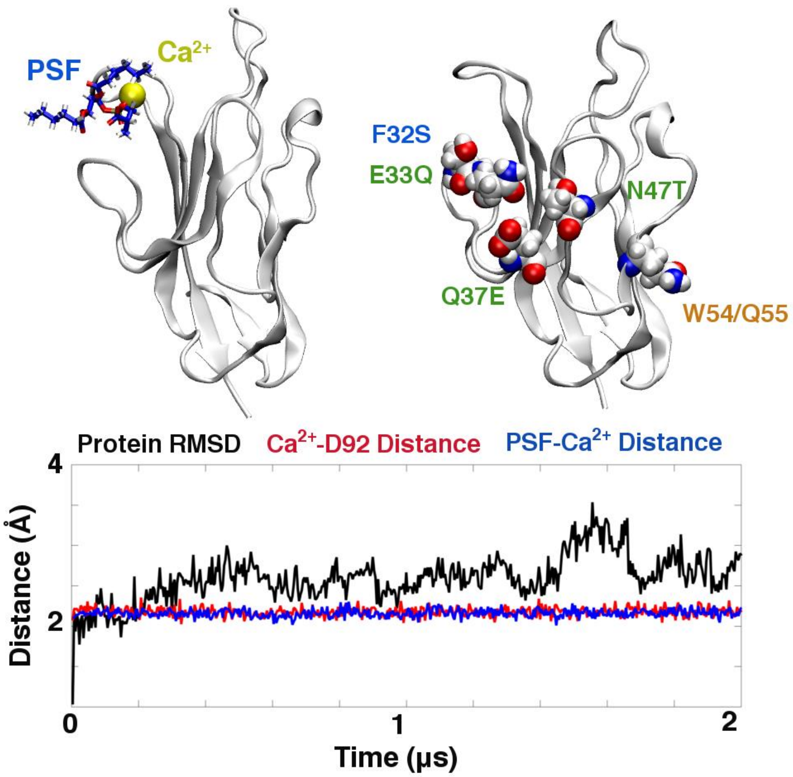

Fig. S6. Illustration of PSF-bound mTIM3 simulations. mTIM3 features several key mutations in relation to hTIM3, with E33Q, Q37E, and Q55/W54 likely being the most significant. PSF and Ca^{2+} remain stably bound to mTIM3 over the course of 2 μs of MD simulation, as the flat red and blue traces indicate. The holo-mTIM3 complex undergoes only a small conformational change over the course of simulation, remaining near 1Å in backbone RMSD from early configurations. Figs. S7 and S8 further demonstrate the similarity between the initial and final states of holo-mTIM3.

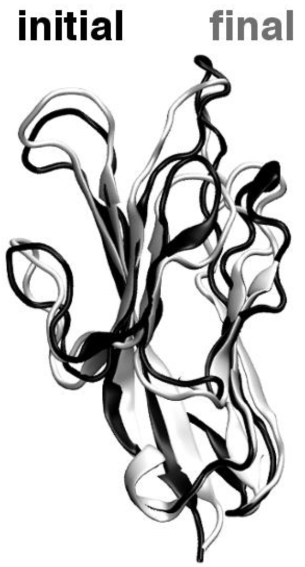

Fig. S7. Overlay of initial and final holo-mTIM3 structures drawn from the 2 μs mTIM3 trajectory. For clarity, PSF is not rendered in either structure. While loop positions fluctuate, no significant conformational change is evident after simulation; the strand shift observed in holo-hTIM3 is not observed in holo-mTIM3.

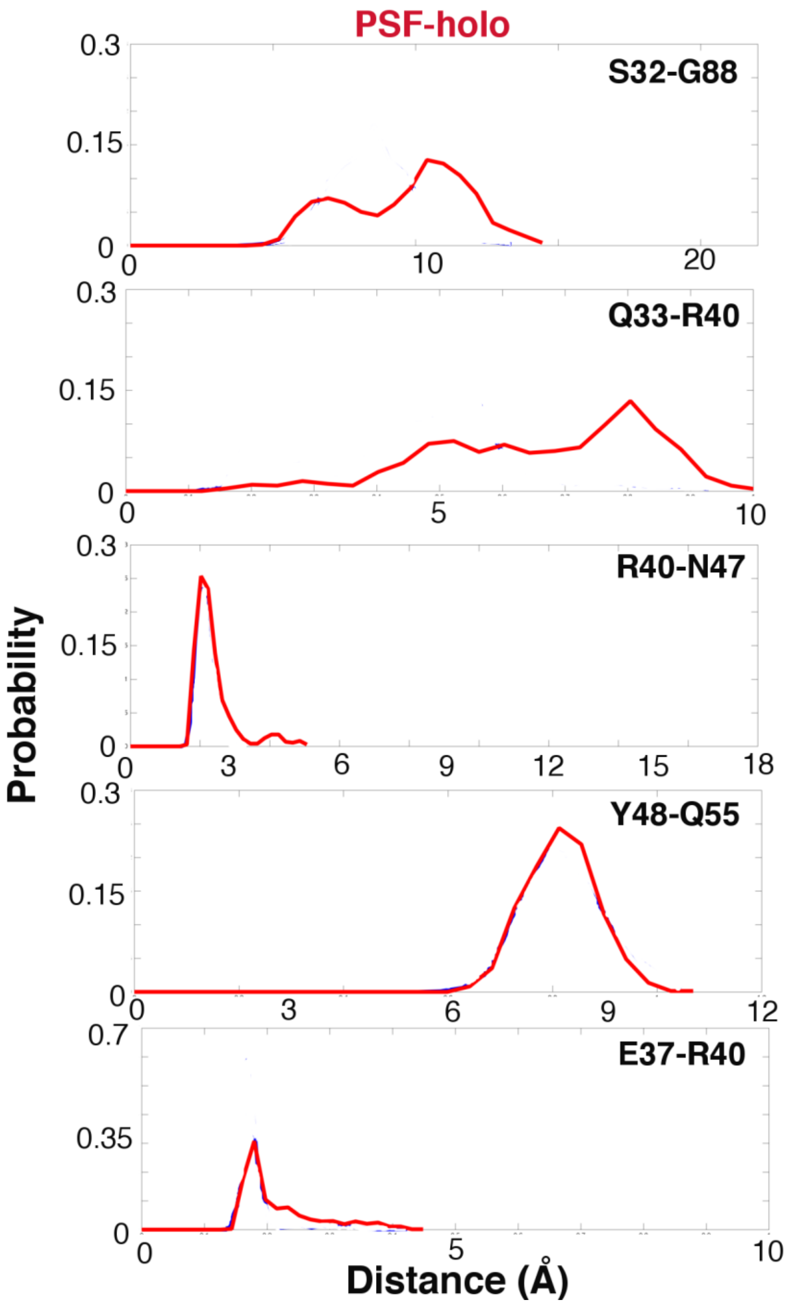

Fig. S8. Residue-residue distance projections of the holo-mTIM3 equilibrium probability distribution, directly comparable to Fig. 4 in the main text. Together, these five projections illustrate that the PSF-induced conformational change observed in holo-hTIM3 does not occur in holo-mTIM3. Notably, E37 of mTIM3 seems to hold R40 in place, and Y48 never comes in contact with Q55 (the homolog of W54 in hTIM3) in mTIM3.
